# Topical Application of Preserved and Unpreserved Bevacizumab Eye Drops Improves Ocular Surface Parameters in Dogs with Chronic Keratitis: A Pilot Study

**DOI:** 10.3390/vetsci13040388

**Published:** 2026-04-17

**Authors:** Ulrike Lessiak, Stefan Kummer, Michael Schueller-Wambacher, Alexander Tichy, Barbara Nell

**Affiliations:** 1 Department of Companion Animals and Horses, University of Veterinary Medicine, 1210 Vienna, Austria; 2 VetCore Facility for Research, University of Veterinary Medicine, 1210 Vienna, Austria; 3 Hospital Pharmacy, University of Veterinary Medicine, 1210 Vienna, Austria; 4 Department of Biomcal Sciences, University of Veterinary Medicine, 1210 Vienna, Austria

**Keywords:** anti-VEGF, OSA-Vet, interferometry, meibography, tear meniscus height, ocular surface

## Abstract

Corneal neovascularization (CNV) in dogs causes chronic ocular surface inflammation, which may disrupt tear film stability and meibomian gland function. As bevacizumab inhibits abnormal vessel growth and affects tear composition, it may benefit dogs with ocular surface disorders. The aim of this pilot study was to evaluate the effects of bevacizumab eye drops on the ocular surface in dogs with chronic CNV. Fourteen eyes from nine client-owned dogs were included. Dogs received either preserved (B_BAC_-group) or unpreserved (B-group) 0.25% bevacizumab eye drops twice daily for 28 days. Re-examinations occurred one week after treatment initiation, at the end of treatment, and three months later. An ocular surface analyzer was used to assess tear composition and meibomian glands via interferometry, tear meniscus height, and meibography. Ocular and systemic toxicities were monitored, and clinical signs were scored. Serum VEGF levels were measured via ELISA before and after treatment. No statistically significant changes in intraocular pressure, tear production, or systemic VEGF levels were observed. Statistically significant improvements were noted in conjunctival hyperemia and chemosis in both groups, ocular discharge in the B_BAC_-group, and fluorescein uptake in the B-group. Both formulations were safe and well tolerated. These preliminary findings suggest that topical bevacizumab may have potential benefits for CNV and ocular surface disorders in dogs, although larger studies are needed.

## Simple Summary

Corneal neovascularization (CNV) in dogs leads to long-lasting inflammation on the eye’s surface and can disturb tear film stability. Because bevacizumab blocks abnormal increase in blood vessel growth, it may help manage these changes. Dogs with chronic CNV received the treatment for four weeks and were examined four times during and after the treatment period. Overall, the medication was well tolerated and safe. Both formulations were associated with improvements in several signs of ocular surface inflammation. The clinical improvements suggest that bevacizumab may support the management of CNV and related surface disease in dogs. These preliminary findings suggest that topical bevacizumab may have potential as a treatment option for dogs with chronic CNV and ocular surface disorders and larger controlled studies are required to confirm these observations.

## 1. Introduction

The transparency of the cornea is essential for its ability to refract light and maintain visual acuity. Under normal physiological conditions, the cornea transmits approximately 99% of incoming light because of its avascular nature and highly organized stromal collagen structure [1]. Transparency is maintained by a complex interplay of antiangiogenic and proangiogenic factors, while an imbalanced expression of angiogenic factors may lead to pathological vascularization, manifesting in two distinct phases; in the initial phase, vascular endothelial growth factor (VEGF), which is predominantly secreted by corneal epithelial cells, binds to VEGF receptors on limbal vascular endothelial cells, initiating angiogenesis [2]. This phase triggers VEGF receptors on limbal vascular endothelial cells to bind with VEGF-A, initiating a proangiogenic cascade that leads to the development of corneal blood and lymphatic vessels [3]. During the secondary phase, the release of proinflammatory cytokines further triggers subsequent angiogenesis [2]. Over time, corneal neovascularization (CNV) compromises the cornea’s transparency, causes edema, and disrupts the corneal immune privilege [4]. This increases the risk of severe complications such as corneal opacities or squamous cell carcinomas [5,6]. Additionally, ocular surface inflammation is closely linked to meibomian gland dysfunction (MGD) in dogs, affecting up to 70% of the examined eyes [7,8].

Anti-VEGF treatments, which were originally developed for retinal diseases in humans, have shown promise in treating vascular-driven ocular diseases, with extensive research validating their efficacy in humans [9,10,11,12,13,14]. Specifically, bevacizumab, a humanized murine anti-VEGF monoclonal antibody, has gained prominence because of its ability to effectively inhibit angiogenesis. Bevacizumab has not only been shown to control CNV but also impacts the precorneal tear film, benefiting other ocular surface disorders, such as MGD and evaporative dry eye (EDE) disease in humans, which are often linked to chronic CNV [15,16,17,18,19]. Hisey et al. [20] underscore the physiological, clinical, and diagnostic similarities between dogs and humans in conditions such as MGD and EDE. These parallels suggest that bevacizumab could be of use for managing these conditions in dogs, supported by their comparable anatomy, functionality, and feasibility of using diagnostic tools originally designed for humans. Furthermore, due to the similarities between canine and human eyes, dogs may also serve as a useful model for exploring the effect of bevacizumab in naturally occurring CNV, tear film instability, MGD, and EDE. A recent study showed that bevacizumab is safe and effective in managing naturally occurring CNV in dogs [21]. Additional studies have also reported successful use of anti-VEGF therapies in managing ocular surface and vascular disorders in veterinary patients [22,23,24]. While bevacizumab eye drops have demonstrated favorable outcomes in managing CNV, treatment with single-dose vials remains economically impractical for routine veterinary use because of increased drug waste, costs and the need for high-volume preparation. To address these limitations, this study also used a formulation of bevacizumab suitable for multidose containers using benzalkonium chloride (BAC) as a preservative [25]. Because preservatives such as benzalkonium chloride may influence ocular surface health and tear film stability, comparing preserved and unpreserved bevacizumab formulations may help determine their relative safety and clinical effects. The impact of bevacizumab eye drops on ocular surface parameters in dogs with CNV has not been previously investigated. The aim of this pilot study was to evaluate the effects of preserved and unpreserved bevacizumab eye drops on ocular surface parameters and the precorneal tear film in dogs with chronic CNV.

## 2. Materials and Methods

### 2.1. Animals and Recruitment Criteria

Client-owned dogs presented at the ophthalmological service of the University of Veterinary Medicine Vienna were enrolled in this study. Adult dogs (>12 months) diagnosed with persistent keratitis and accompanying superficial or deep corneal vascularization despite topical treatment with cyclosporin A (CYA) and/or corticosteroids (DEX) for at least 28 days were included. At the enrollment visit (T0), animals were excluded if their Schirmer tear test-1 (STT-1) measurements were less than 15 mm/min, if known coagulation disorders were present or if the animal showed clinical manifestations of systemic disorders. The signalment and medical history were recorded for each dog as indicated by the owner. The study was approved by the institutional ethics and animal welfare committee and the national authority according to §§ 26ff. of Animal Experiments Act, Tierversuchsgesetz 2012—TVG 2012 (GZ 2021-0.391.968). Informed owner consent was obtained from all owners as part of the enrollment procedure.

### 2.2. Drug Preparation

A 0.25% solution of bevacizumab eye drops was aseptically prepared from a commercially available bevacizumab solution (Avastin^®^, Roche Pharma AG, Grenzach-Wyhlen, Germany) by the institutional pharmacy according to good manufacturing practices. Sterile 0.9% saline solution (B. Braun, Melsungen, Germany) was used as the solvent. The study medication was provided in single-dose containers for each dog with either preserved bevacizumab (0.5 mL of 0.25% bevacizumab + 0.01% benzalkonium chloride, B_BAC_-group) or unpreserved bevacizumab (0.5 mL of 0.25% bevacizumab, B-group). With regard to the shelf-life of bevacizumab, the eye drops were prepared after the study enrollment (T0) and stored by the owner protected from light at 2–8 °C [25,26]. The physicochemical properties, stability, and biological activity of the preserved and unpreserved bevacizumab formulations used in this study have been comprehensively characterized in a previous in vitro investigation [25]. The content of the containers was masked by the pharmacist (MSW) and only revealed after the end of the study and completion of analysis of the obtained data. A detailed written instruction leaflet was enclosed.

### 2.3. Study Design

This prospective, randomized, single masked, proof-of-concept pilot study was designed to compare the effects and safety of preserved and unpreserved formulations of bevacizumab (Avastin^®^) eye drops on the ocular surface of dogs with persistent CNV. This study was designed as an exploratory, hypothesis-generating investigation to inform future trials. The dogs were treated and examined on an outpatient basis, with eyedrops administered by the owners after receiving detailed instructions. The eyes were randomized in a 1:1 ratio to receive either preserved bevacizumab (B_BAC_-group, 0.25% bevacizumab eyedrops containing 0.01% BAC) or unpreserved bevacizumab (B-group, 0.25% bevacizumab eyedrops) administered at 12-h intervals for 28 days. In cases where both eyes of a dog met the inclusion criteria, each eye was treated and analyzed as an individual unit. Treatment was initiated within one week after the enrollment visit (T0) due to the manufacturing of the study medication. Re-examinations were scheduled 1 week after treatment initiation (T1), at the end of the 28-day treatment period (T2) and 3 months after the end of the treatment period (T3). If necessary, additional follow-up examinations were scheduled at the discretion of the study investigators. Prior to initiation of the study, each dog underwent a full physical and ocular examination, and a complete blood count, including hematology, baseline serum VEGF values, activated partial thromboplastin time, partial thromboplastin time, and thrombin time, was conducted to ensure eligibility. Each study visit included an assessment of ocular and systemic effects and pain assessment, as described below. Serum VEGF levels were measured at baseline (T0) and at the end of treatment (T2) to monitor for any potential systemic exposure, given that certain topical ophthalmic drugs can undergo measurable systemic absorption in dogs and that preservatives such as benzalkonium chloride can increase penetration of active compounds [27,28]. A detailed schedule of interventions is shown in Table 1.

### 2.4. Assessment of Ocular Parameters

#### 2.4.1. Ocular Surface Examination

**Figure 1:**
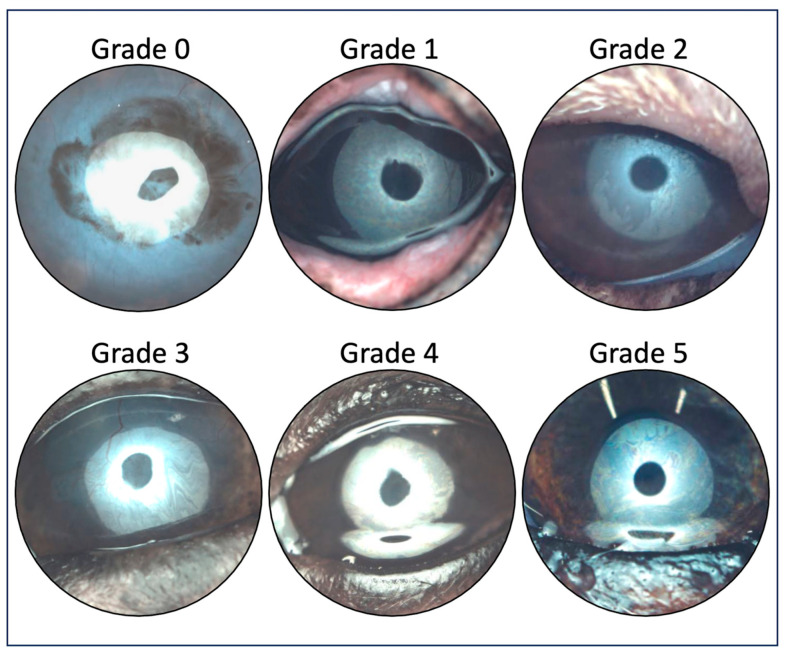
Interferometry score system by Jeong et al. [29] with representative pictures of the study cohort: Grade 0 = absence of a lipid layer; Grade 1 = spaced meshwork patterns with gray or marble-like colors, the iris is still visible; Grade 2 = more closed meshwork patterns with occasional gray or marble-like waves; Grade 3 = constant wave or fluid-like pattern with almost-straight lines; Grade 4 = amorphous and homogenous pattern with bright (white bright/yellowish) colors, the iris is not visible; Grade 5 = color fringe pattern formed by waves of yellow, brown, purple and blue.

The Ocular Surface Analyzer (OSA-Vet, SBM Sistemi, Rivalta di Torino, Italy) was used for detailed structural examination of the precorneal tear film and meibomian glands (MGs). Interferometry was carried out by observing lipid layer patterns formed 2–3 s after complete manual blinking. For each measurement, the OSA-Vet was positioned before the eyelids were opened. A complete manual blink was then induced by the examiner, and an image was captured as soon as the eye was opened and the device was in focus. Multiple images were obtained per eye, and the interferometry score was assessed based on the most representative image. A six-point scoring system was used, as described by Jeong et al. [29] (Figure 1): grade 0 = absence of a lipid layer; grade 1 = spaced meshwork patterns with gray or marble-like colors, in which the iris is still visible; grade 2 = more closed meshwork patterns with occasional gray or marble-like waves; grade 3 = constant wave or fluid-like pattern with almost straight lines; grade 4 = amorphous and homogenous pattern with bright (white bright/yellowish) colors, in which the iris is not visible; grade 5 = color fringe pattern formed by waves of yellow, brown, purple and blue waves.

**Figure 2:**
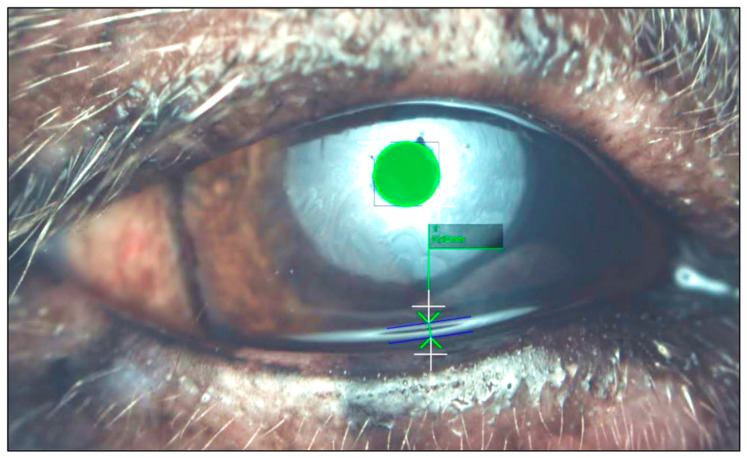
Measurement of tear meniscus height (TMH). Representative image of TMH measurements obtained by comparing the diameter of the inner circle of the light circle with the distance from the upper limit to the lower limit of the inferior tear meniscus. TMH was calculated in mm using the OSA-Vet program.

In addition, the tear meniscus height (TMH) was examined, by measuring the distance between the upper and lower limit at the midpoint of the inferior tear meniscus (Figure 2). Each measurement was taken immediately after a spontaneous blink in triplicates via the program’s software (OSA-Vet, SBM SistemiSystems).

**Figure 3:**
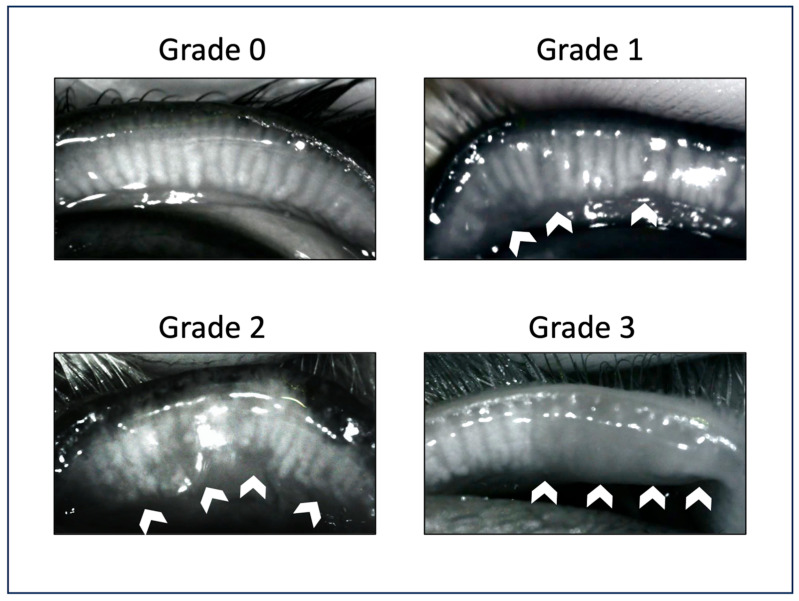
Meibography scoring system by Arita et al. (2013) with representative pictures of the study cohort: Grade 0 = normal meibomian glands without dropouts; Grade 1 = loss of up to one-third of the meibomian glands (white arrows); Grade 2 = loss of more than one-third and less than two-thirds of the meibomian glands (white arrows); Grade 3 = loss of more than two-thirds of the meibomian glands. Note the generalized darkened area with no normal gland morphology (white arrows).

Infrared meibography was used to assess changes of morphology and dropout of the MGs through eversion of the middle two-thirds of the upper and lower eyelid. The obtained infrared pictures were analyzed using the grading system previously described by Arita et al. [30] (Figure 3): grade 0 = no apparent MG loss; grade 1 = dropout of less than one -third of MGs; grade 2 = dropout of more than one-third and less than two-thirds of MGs; and grade 3 = dropout of more than two-thirds of MGs.

#### 2.4.2. Ocular Examination

A complete ophthalmic examination, including slit-lamp biomicroscopy (Kowa SL-15; Kowa, Aichi, Japan), indirect ophthalmoscopy (Keeler Vantage; Keeler Instruments Inc., Malvern, PA, USA), Schirmer tear test-1 (MSD), fluorescein staining (Fluorotouch Ophthalmic Strips, Eickemeyer, Tuttlingen, Germany), and measurement of the intraocular pressure using rebound tonometry (TonoVet Icare, Vantaa, Finland), was carried out in all dogs by the same investigator (UL) under the supervision of a board-certified ophthalmologist (BN). A minimum of 10-min intermission between the OSA-Vet assessment and the ophthalmic examination was allowed to ensure unspoiled results [31]. Posterior eye segment examination was conducted after the induction of pharmacological mydriasis (Tropicamid, Mydriaticum, and Agepha) at study enrollment (T0) and at the last follow-up examination (T3). Macroscopic findings regarding conjunctival hyperemia, conjunctival chemosis, ocular discharge and fluorescein uptake were quantified using a modified Hackett-McDonald scoring system: 0 = none, 1 = mild, 2 = moderate, and 3 = severe [21,32].

### 2.5. Assessment of Systemic Parameters

#### 2.5.1. Physical Examination

A complete physical examination was conducted at each timepoint. Monitoring of vital parameters, including respiratory and heart rate, mucous membrane color and capillary refill time, was performed. Dogs with a history of allergies were specifically examined regarding possible flare-ups.

#### 2.5.2. Serum VEGF Values

Obtained blood samples were allowed to rest for at least 30 min after collection before being centrifuged for 20 min at 1000× *g* and the serum supernatant was stored in sterile polypropylene tubes at −80 °C. A commercially available canine enzyme-linked immunosorbent assay (ELISA) was used to measure serum VEGF values (Canine VEGF Quantikine ELISA Kit, R&D Systems, Minneapolis, MN, USA). The measurements were conducted in accordance with the assay instructions. Standards, samples, and assay diluents were added to each well and incubated for 2 h at room temperature. After washing, the wells were incubated with the VEGF conjugate for 2 h at room temperature. Color substrate was added to each well, and color development was stopped after 25 min. Absorbance at 450 nm was quantified in a microplate reader (Promega Corporation, Madison, WI, USA). The standard curve was obtained by serial dilutions of VEGF-A. All standards and samples were analyzed in triplicates.

### 2.6. Pain Assessment

Continuous monitoring for ocular and systemic toxicity was assessed using a modified pain scores system [33]. Any adverse event reported by the owner, or abnormal ocular or general finding observed during the study rechecks were recorded. Pain scoring categories included (1) eye comfort, indicated by the degree of blepharospasm, blinking, scratching and/or tearing; (2) unprovoked behavior; and (3) interactive behavior, indicated by the reaction when the eye surroundings were touched. The modified pain scores system can be found as Supplementary Table S3. Pain scores were recorded at each time point by the same investigator (UL). Additionally, the owners were asked to evaluate pain sensation 6 h and 3 days after treatment initiation.

### 2.7. Data Analysis

Randomization was performed using Microsoft Excel. Each study medication was assigned a four-digit random number and a corresponding group allocation (green = B_BAC_; red = B). The dataset was then randomized based on these assigned random numbers to generate the final allocation sequence. The findings of the ophthalmic examination were documented in the facilities own data software system. The ocular surface parameters were documented in the OSA-Vet software program and exported to an excel file. Additional data collection and analysis were performed using Microsoft Excel (Microsoft Corporation, Microsoft Excel 2016). The values for both treatment groups were listed as mean ± standard deviation (SDs) and 95% confidence interval (CIs) were feasible. Metric-scaled values obtained at different time points were analyzed using linear mixed-effects models in which group, timepoint and the interaction term were added as fixed factors to the model. Metric-scaled values obtained at different time points were analyzed using linear mixed-effects models, including group, timepoint, and their interaction as fixed effects. Multiple comparisons were performed using Sidak’s alpha correction procedure. Ranked data were analyzed using the Wilcoxon test. All analyses were performed using IBM SPSS Statistics for Windows, Version 29.0. A *p*-value below 5% (*p* < 0.05) was considered statistically significant. Missing data were not imputed, and analyses were performed using available-case data. All data were analyzed at the level of the individual eye unless otherwise specified. The extent of missing data varied between parameters, due to anatomical and compliance-related limitations. Missing data were handled using a complete-case approach. No imputation was performed, in line with the exploratory design of this pilot study.

## 3. Results

### 3.1. Animals

Eighteen eyes of 11 dogs met the inclusion criteria and were included in the study. Study visits were conducted at 7 ± 2 days (T1), 28 ± 5 days (T2) and 115 ± 10 days (T3) after treatment initiation. Two dogs were excluded after the first follow-up visit (T1) due to lack of owner compliance, resulting in the loss of four eyes. Of the remaining nine dogs, five were male castrated (5/9, 55.6%), two dogs were female and spayed (2/9, 22.2%), and two dogs were male (2/9, 22.2%), with a mean weight of 13.4 ± 11 kg and a mean age of 6.4 ± 3.3 years. The breeds included in the study consisted of two Chihuahua dogs, two French Bulldogs and one American Stafford Terrier, English Bulldog, Havanese, Papillon, and mixed breed each, resulting in five brachycephalic (5/9, 55.6%) and four mesocephalic dogs (4/9; 44.4%). The signalment and corneal diagnosis of the two groups are listed in Table 2; detailed patient history and pretreatment information are shown in (Supplementary Tables S1 and S2). Five dogs were treated bilaterally, of which two dogs were treated with preserved eye drops in one eye and unpreserved eye drops in the other, one dog was treated with preserved bevacizumab bilaterally and two dogs with unpreserved bevacizumab bilaterally. The exclusion of four eyes resulted in an uneven ratio within the two groups, with five eyes of four dogs (5/14 eyes, 35.7%) included in the B_BAC_-group and nine eyes of seven dogs (9/14 eyes, 64.3%) included in the B-group. Three right (3/14 eyes, 21.4%) and two left eyes (2/14 eyes, 14.3%) received preserved bevacizumab eye drops, and six left (6/14, 42.9%) and three right eyes (3/14 eyes, 21.4%) received unpreserved bevacizumab eye drops.

### 3.2. Ocular Parameters

Mean intraocular pressure (IOP) measurements and STT-1 values remained within normal limits, with minimal variations except for one dog (dog #F/eyes 10,11, B-group). At the long-term follow-up examination (T3), the dog showed a KCS flare-up with decreased STT-1 values (OD 11 mm/min and OS 14 mm/min) despite its long-term treatment with topical cyclosporine (Optimmune^®^ 0.2%, Merck & Co), dexamethasone dihydrogen phosphate (Monodex^®^ 1 mg/mL, Théa Pharma) and systemic pilocarpine 1% (prepared by the hospital pharmacy of the University of Veterinary Medicine, Vienna). Tear production was unresponsive to a subsequent increase of the cyclosporin concentration (cyclosporin 2%). As the owner reported that the dog had been more comfortable during the study period, an additional 28-day treatment period with bevacizumab eye drops was initiated. Tear production was normalized within six days of treatment, and no relapse was reported until the last check-up.

There was no statistically significant difference between the mean STT-1 and IOP values before treatment (T0) and the subsequent follow-up examinations (T1, T2, T3). Mean ± SD of the intraocular pressure measurements and STT-1 values are shown in Table 3.

**Figure 4:**
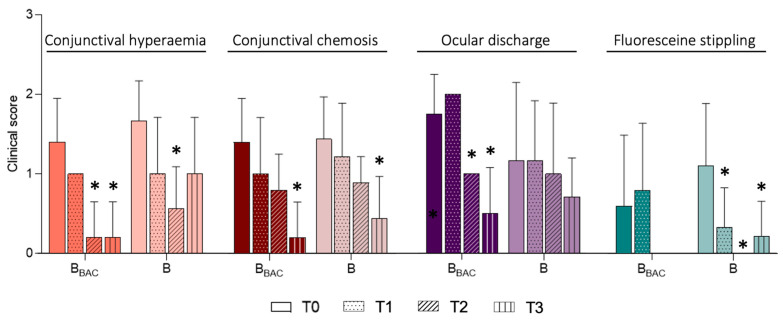
Macroscopic ocular findings. The scores for conjunctival hyperemia (0–3), conjunctival chemosis (0–3), ocular discharge (0–3) and fluorescein stippling (0–3) before (T0), 7 days (T1), 28 days (T2) and 4 months (T3) after treatment initiation are shown as the means ± SDs. Grade 0 = none; Grade 1 = mild; Grade 2 = moderate; Grade 3 = severe. Fluorescein stippling refers to minimal multifocal punctuate fluorescein uptake. B_BAC_ (n = 5) and B (n = 9). The asterisk (*) indicates statistically significant differences from T0.

Overall, the mean scores evaluated for ocular findings decreased in both groups over the 28-day treatment period (Figure 4). Specifically, a statistically significant reduction was observed for conjunctival hyperemia in both groups for ocular discharge in the B_BAC_-group, and for fluorescein stippling in the B-group. Furthermore, reduced scores were obtained for conjunctival chemosis in both groups, for ocular discharge in the B-group and for fluorescein stippling in the B_BAC_-group, although the differences were not statistically significant. Additionally, the clinical effect was sustained and observed even during the long-term follow-up examination (T3), with conjunctival hyperemia scores of 0.2 ± 0.5 (B_BAC_) and 1.0 ± 0.71 (B), conjunctival chemosis scores of 0.2 ± 0.5 (B_BAC_) and 0.4 ± 0.5 (B), ocular discharge scores of 0.6 ± 0.6 (B_BAC_) and 0.8 ± 0.4 (B) and fluorescein stippling scores of 0.0 ± 0.0 (B_BAC_) and 0.2 ± 0.4 (B). Scores are shown in Table 4.

**Figure 5:**
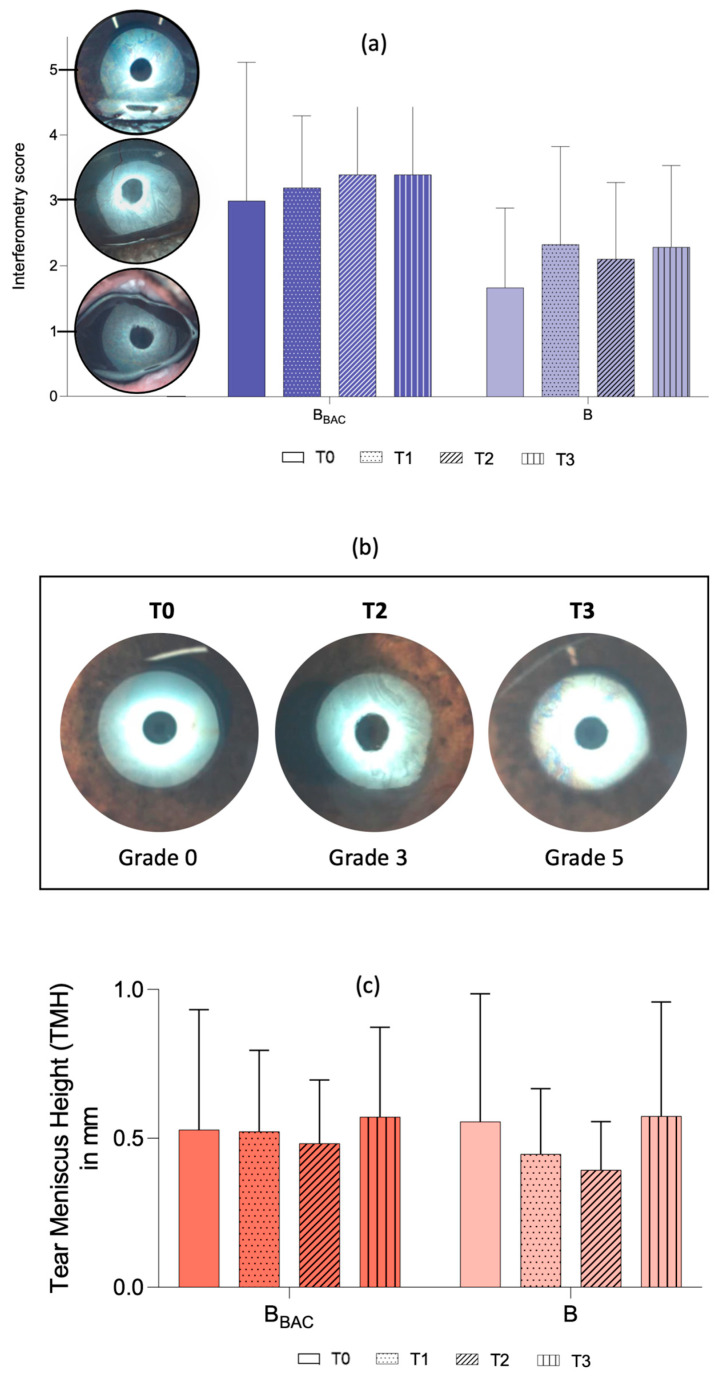
Interferometry and tear meniscus height (TMH). (**a**) Interferometry scores are shown as mean ± SD before (T0), 7 days (T1), 28 days (T2) and 4 months (T3) after treatment initiation with either preserved (B_BAC_) or unpreserved (B) bevacizumab. (**b**) Representative images of one eye (#14, B-group) showing increasing interferometry scores over the study period. Note the homogenous surface at T1; the wave or fluid-like pattern at T2 and the waves of yellow, brown, purple and blue at T3. (**c**) Tear meniscus height is shown as the mean ± SD (in mm) before (T0), 7 days (T1), 28 days (T2) and 4 months (T3) after treatment initiation with either preserved (B_BAC_) or unpreserved (B) bevacizumab.

Lipid layer thickness assessed with interferometry remained largely stable throughout the study period, with a slight, non-significant increase observed in both groups (Table 5, Figure 5).

Mean TMH remained stable, with minimal variations in both groups over the study period. TMH values slightly decreased in eyes treated with unpreserved bevacizumab during the 28-day treatment period but ultimately increased again when measured at the long-term follow-up examination (Table 6, Figure 5).

**Figure 6:**
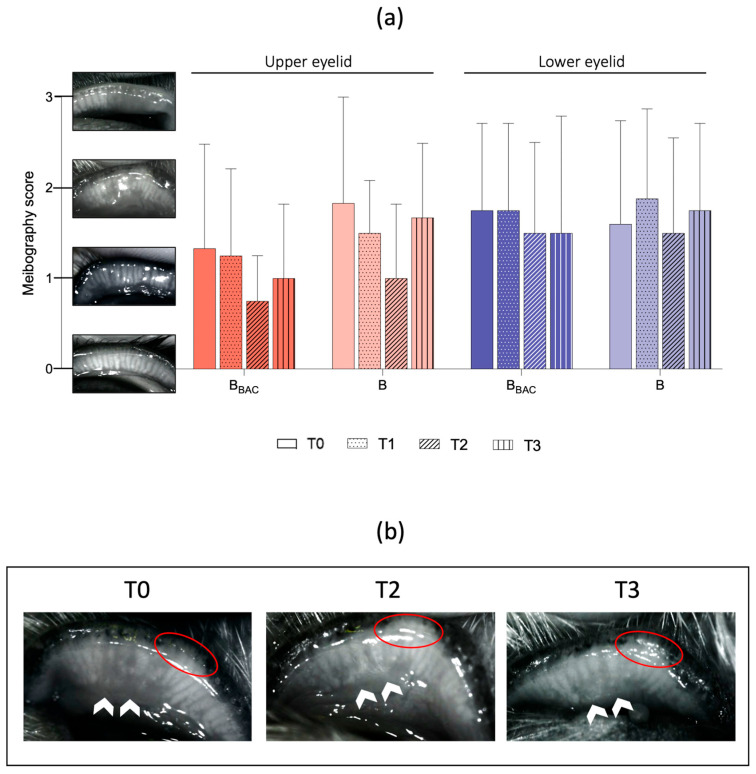
Meibography. (**a**) Scores are shown as mean ± SD for the upper and lower eyelid before (T0, B_BAC_: n = 7, B: n = 11), 7 days (T1, B_BAC_: n = 7, B: n = 12), 28 days (T2, B_BAC_: n = 8, B: n = 13) and 4 months (T3, B_BAC_: n = 8, B: n = 13) after treatment initiation with either B_BAC_ or B. A statistically significant decrease in gland dropout was noticed in both groups during the treatment period, with an increase to baseline at lo 7 ng-term follow-up. (**b**) Representative images of one eye (#1, B_BAC_-group) with decreasing gland dropouts (areas of interest marked with white arrows) over the study period. Notice the lid depigmentation (red circles) as a reference point.

Due to either pigmentation of the conjunctiva, anatomical conformation, or lack of patients’ compliance, infrared meibography images of either the upper (UL) or lower (LL) eyelid could be taken in 18 (18/28, 64%) at T0, 19 (19/28, 68%) at T1 and 21 (21/28, 75%) eyelids at T2 and T3 each. At the initial examination (T0), the mean meibography scores of UL and LL were 1.6 ± 1.0 and 1.7 ± 1.1 for eyes in the B_BAC_- group and in the B- group, respectively. The meibography scores remained stable throughout the study period, with a slight decrease in MG dropout in both groups at the end of the treatment period to 1.1 ± 0.8 in the B_BAC_ group and 1.2 ± 0.9 in the B group, although without statistically significance (Table 5, Figure 6).

### 3.3. Systemic Impact

No clinical signs of systemic incompatibility or adverse events were noted in any dog at any time point. All values obtained from the physical examination remained within the normal clinical range with minimal variations. One dog (#D/5, B_BAC_- group) with a known history of atopic dermatitis developed otitis externa bilaterally, which was successfully treated with locally applied INN-Terbinafine-Florfenicol-Betamethasone-Acetateterbinafine-florfenicol-betamethasone-acetate (Osurnia^®^, Dechra Pharmaceuticals plc.) after cytological confirmation. The same dog was presented at the emergency care unit due to an abscess of the anal gland 11 days after discontinuation of the topical bevacizumab treatment.

There was no statistically significant change in the mean serum VEGF values before and after 28 days of treatment in both groups (Table 7).

### 3.4. Pain Assessment

Changes in behavior, touch response and/or eye comfort after the first administration of bevacizumab were reported by two owners (dog #C/eyes 4,7, B_BAC_- and B-group; dog #E/eyes 8,9, B-group). The owner of dog #I (eye 14, B-group) noticed increased rubbing and cloudiness of the treated eye 27 days after treatment initiation. On the same day, no signs of ocular discomfort or abnormalities could be observed during the ophthalmic examination. In all other dogs, there was no evidence of ocular or systemic pain associated with bevacizumab eye drops. In contrast, owners reported increased visual acuity (dog #A/eyes 1,6, B_BAC_- and B-group; dog #F/eyes 10,11, B-group), less rubbing (dog #H/eye 13, B-group) and/or less cloudiness (dog #H/eye 13, dog #I/eye 14, B-group) of the treated eyes during the 28-day study period. One dog (dog #C/eyes 4, and 7, B_BAC_- and B-group) showed mild depression at study initiation (unprovoked behavior). However, none of the dogs showed any change in behavior at study visit T1, T2 and T3.

Pain scores assessed by the investigator (UL) showed that the drug was safe and well tolerated throughout the study period (Table 8).

## 4. Discussion

This pilot study suggests potential beneficial effects of both preserved and unpreserved bevacizumab eye drops on ocular surface parameters in dogs with therapy-resistant chronic CNV. Interestingly, assessment of meibomian gland morphology showed a reduction in the degree of gland dropout after treatment with both preserved and unpreserved bevacizumab. Although without statistically significance, this might suggest the potential for enhancing meibomian gland function. Despite controversial evidence on gland dropout reversibility, previous studies reported a decrease in dropout rates of approximately 5% after MGD treatment, such as eyelid warming, hygiene and topical therapy in humans [30,34,35]. However, given that meibomian gland dropout is generally considered a structural change unlikely to reverse within a short treatment period, and that several meibography images were unavailable due to pigmentation, conformation, or patient compliance, these findings must be interpreted with caution. As analyses were based on available-case data without imputation, missing data may not be random and could introduce bias.

Interferometry showed a slight increase in lipid layer thickness following treatment in both groups. Although not statistically significant, these findings may suggest that bevacizumab eye drops may have a favorable effect on the tear film. While a consistent direction of change was observed, the lack of statistical significance limits the ability to draw conclusions regarding efficacy. The findings align with previous reports of improved tear film stability in humans with MGD following bevacizumab administration [15,18]. One possible explanation for the increased tear film stability could be the positive impact of bevacizumab on ocular inflammation [36], as evidenced by decreased conjunctival hyperemia and chemosis, ocular discharge, and fluorescein staining scores in both treatment groups. These improvements were observed as early as one week after treatment initiation and were sustained throughout the 28-day treatment period, with further enhancement noted at the long-term follow-up examination, although the findings should be interpreted cautiously given the small sample size.

Studies have demonstrated that inflammation plays a key role in EDE pathogenesis, with increased levels of proinflammatory cytokines, such as interleukin (IL-1, IL-6, and IL-8), tumor necrosis factor-alpha (TNF-α), interferon-gamma (INF-γ) and matrix metalloproteinase (MMP-9), detected in the tear film and conjunctival epithelium of affected individuals [36,37,38,39,40,41,42]. VEGF-A, which acts as a proinflammatory factor, stimulates the release of these factors and promotes pathological events in MGD and EDE disease [14,39]. Furthermore, VEGF-A expression is increased in inflamed corneas, and anti-VEGF agents have been shown to significantly decrease ocular inflammation compared with dexamethasone in a murine model [15,43,44]. Moreover, the duration and progression of a disease can impact the temporal expression of angiogenic factors. For example, in models of dry eye disease (DED), VEGF-D expression initially increases, whereas VEGF-A and VEGF-C expression occurs at later stages of the disease [45]. The interferometry results provide additional insight into tear film dynamics but warrant careful consideration. The visual differences between interferometry images can be subtle and may be difficult to appreciate in representative figures alone. Therefore, assessment was based on a standardized and previously described grading system [29]. In addition, variability in image acquisition, tear film dynamics, and patient cooperation may have influenced the results.

The transient KCS flare-up in one dog at the long-term follow-up is most likely attributable to the dog’s underlying predisposition and chronic disease course, as the flare-up occurred after the treatment period and resolved rapidly once bevacizumab was re-introduced. Bevacizumab has previously shown beneficial effects on ocular surface disease in human patients with dry eye, including improvements in ocular surface disease index (OSDI) scores and STT-1 values [17,19]. Nonetheless, this was an isolated case and no conclusions regarding potential effects of bevacizumab on KCS in dogs can be drawn from a single observation.

While this pilot study utilized a prospective, randomized, single-mask design, limitations should be considered. First, the sample size was small, which limits the validity of the findings. Several outcomes showed descriptive trends without reaching statistical significance, and these should be interpreted as observational findings rather than evidence of a treatment effect. Furthermore, the unequal distribution of eyes between the two treatment groups could introduce bias in the analysis and interpretation of the results. Future studies with larger sample sizes should provide more robust evidence. Given that some dogs contributed both eyes to the analysis, this may introduce intra-individual correlation, potentially affecting the independence of observations and leading to an underestimation of variability. Given the small sample size, this approach was chosen to maximize the available data. Furthermore, the dogs in this study displayed significant variability in the extent of corneal scarring and blood vessel depth, which are crucial for drug penetration [46]. Due to the small sample size, subgroup analyses were not considered statistically meaningful. Future studies could benefit from grouping patients based on the extent of corneal vascularization and scarring to investigate correlations with drug efficacy. This study was not designed to quantify changes in CNV, as the anti-angiogenic effect of bevacizumab has already been well established in both veterinary and human ophthalmology [9,10,11,12,13,14,21,22,23,24].

Additionally, this study did not include a control group. The enrolled population consisted of client-owned dogs with long-standing, treatment-refractory disease, so discontinuation or withholding of established therapy was not considered ethically justifiable. As a result, a placebo-controlled design was not feasible in this clinical setting. All dogs had received prolonged prior treatment without clinical improvement and exhibited stable disease before study inclusion, which supports the assumption that observed changes were unlikely due to spontaneous resolution. Moreover, the primary objective of this pilot study was to determine whether the preserved formulation (B_BAC_) could be used without clinical disadvantage compared to the unpreserved formulation (B), thereby supporting a more accessible and cost-effective treatment option for clinical practice. Nonetheless, we acknowledge that the absence of a placebo group represents a limitation of the study, and future randomized controlled trials with larger cohorts are warranted to confirm these findings.

Although several owners reported impressions of improved visual acuity or reduced cloudiness, these subjective observations should be interpreted with caution, as they may not correspond to measurable clinical changes. In addition, the applied pain scoring system included behavioral parameters (spontaneous and provoked behavior) as well as signs of ocular discomfort such as blepharospasm, blinking, and scratching. While a reduction in pain scores was observed over time, behavioral components are susceptible to external influences, including habituation to the clinical environment, increased familiarity with the examiner, and repeated handling. Additionally, improved compliance with topical medication during the study period may have contributed to reduced sensitivity of the periocular region. Therefore, pain-related findings should be interpreted within the context of these limitations.

The current study conducted follow-up examinations up to 3 months after the end of the treatment period, providing insights into the long-term effects of bevacizumab. However, maintenance of clinical improvement has been reported to last as long as 6 months after treatment in dogs and humans with CNV [11,12,21]. This underscores the potential for prolonged efficacy beyond the treatment period. Recent findings by [47] revealed the significant influence of cranial features on ocular surface parameters, particularly in brachycephalic dogs. Their research demonstrated that these dogs present shorter TBUTs, thinner lipid layers, and higher rates of meibomian gland loss than other cephalic types do. Although our study did not focus specifically on head conformations, 2/4 (50%) and 3/7 (42.8%) dogs in the B_BAC_- and B-groups, respectively, were brachycephalic. Future research should consider head conformations.

## 5. Conclusions

In conclusion, this pilot study provides preliminary evidence supporting the positive effect of preserved and unpreserved bevacizumab eye drops on the ocular surface in dogs with naturally occurring CNV. These findings suggest that bevacizumab therapy may have beneficial effects not only for treating corneal vascularization but also for reducing ocular surface inflammation and improving tear film stability and meibomian gland function. Further research involving larger cohorts, optimal dosing regimens, treatment duration, and longer follow-up periods is warranted to corroborate these preliminary findings.

## Figures and Tables

**Table 1:** Study design.

	T0	6 h	3 d	T1	T2	T3
	Study enrollment	6 h after treatment initiation-conducted by owner	3 d after treatment initiation–conducted by owner	1st follow-up visit 1 week after treatment initiation	2nd follow-up visit at end of 28-day treatment period	3rd follow-up visit 3 months after end of treatment period
Physical examination	✔			✔	✔	✔
Ophthalmological examination before and after pharmacological mydriasis ^a^	✔					✔
Ophthalmological examination without pharmacological mydriasis ^a^				✔	✔	
Pain assessment	✔	✔	✔	✔	✔	✔
Complete blood count ^b^	✔					
Serum VEGF-level measurement	✔				✔	
Corneal photography	✔			✔	✔	✔
OSA Vet analysis ^c^	✔			✔	✔	✔

^a^ including slit-lamp biomicroscopy, indirect ophthalmoscopy, Schirmer tear test-1, fluorescein staining. ^b^ consists of hematology, activated partial thromboplastin time, partial thromboplastin time, and thrombin time. ^c^ consists of measurement of tear meniscus height, interferometry and meibography.

**Table 2:** Signalment and corneal findings of the studied dogs.

Patient	Eye #		Breed	Age in Years	Gender	Weight in kg	Corneal Diagnosis
B_BAC_							
A *	1	OD	Havanese	13	MC	5.7	Superficial keratitis, pigmentary keratitis
B *	2	OD	Chihuahua	5	FC	3.9	Superficial keratitis, corneal fibrosis
	3	OS		5	FC	3.9	Superficial keratitis, corneal fibrosis
C *	4	OS	American Staffordshire Terrier	4	MC	26	Punctate keratitis
D	5	OD	Chihuahua	2	M	3.6	Superficial stromal keratitis, corneal fibrosis
B							
A *	6	OS	Havanese	13	MC	5.7	Superficial keratitis, pigmentary keratitis, corneal fibrosis
C *	7	OD	American Staffordshire Terrier	4	MC	26	Punctate keratitis
E *	8	OD	English bulldog	4	MC	27.9	Superficial stromal keratitis, corneal fibrosis
	9	OS		4	MC	28.9	Superficial stromal keratitis
F *	10	OD	Papillon	6	FC	2.85	Superficial stromal keratitis, pigmentary keratitis, corneal fibrosis
	11	OS		6	FC	2.85	Superficial stromal keratitis, pigmentary keratitis
G	12	OS	French bulldog	9	MC	12.4	Superficial stromal keratitis, pigmentary keratitis, corneal fibrosis
H	13	OS	French bulldog	7	MC	10.9	Stromal keratitis, pigmentary keratitis, corneal fibrosis
I	14	OS	Mixed breed	8	M	26.7	Superficial keratitis

* bilaterally treated, OS = Oculus sinister, OD = oculus dexter, FC = female castrated, M = male, MC = male castrated.

**Table 3:** Mean ± SD of STT-1 and IOP values of the studied dogs at different timepoints (T0, T1, T2, and T3).

	T0		T1			T2			T3		
STT-1	mm/min	95% CI	mm/min	95% CI	*p*-value	mm/min	95% CI	*p*-value	mm/min	95% CI	*p*-value
B_BAC_ (n = 5)	23.8 ± 2.4	21.7–25.9	22.4 ± 3.4	19.5–25.3	0.53	22.6 ± 1.1	21.6–23.6	0.61	21 ± 2.5	18.8–23.2	0.29
B (n = 9)	23.6 ± 4.3	20.7–26.4	21.3 ± 2.9	19.4–23.2	0.19	22.6 ± 4.3	19.7–25.4	0.57	20.2 ± 5.0	16.9–23.5	0.10
IOP	mmHg	95% CI	mmHg	95% CI	*p*-value	mmHg	95% CI	*p*-value	mmHg	95% CI	*p*-value
B_BAC_ (n = 5)	21.0 ± 3.5	17.9–24.1	19.6 ± 2.5	17.4–21.8	0.43	18.6 ± 3.2	15.8–21.4	0.19	21.8 ± 1.9	20.1–23.5	0.62
B (n = 9)	17.8 ± 2.0	16.5–19.1	19.4 ± 3.1	17.4–21.5	0.21	20.0 ± 2.9	18.1–21.9	0.11	18.8 ± 2.6	17.1–20.5	0.41

CI = Confidence interval, IOP = Intraocular pressure, and STT = Schirmer-Tear Test.

**Table 4:** Mean ± SD of conjunctival hyperemia, conjunctival chemosis, ocular discharge and fluorescein stippling grades of the studied dogs at different timepoints (T0, T1, T2, and T3).

	T0	T1		T2		T3	
conjunctival hyperemia							
B_BAC_ (n = 5)	1.4 ± 0.6	1.0 ± 0.0	*p = 0.09*	0.2 ± 0.45	p < 0.01 *	0.2 ± 0.5	p = 0.02 *
B (n = 9)	1.7 ± 0.5	1.0 ± 0.7	p < 0.01 *	0.6 ± 0.5	p < 0.01 *	1.0 ± 0.7	p = 0.03 *
conjunctival chemosis							
B_BAC_ (n = 5)	1.4 ± 0.6	1.0 ± 0.7	*p = 0.19*	0.8 ± 0.5	*p = 0.10*	0.2 ± 0.5	p < 0.01 *
B (n = 9)	1.4 ± 0.5	1.22 ± 0.7	*p = 0.09*	0.9 ± 0.3	p = 0.01 *	0.4 ± 0.5	p < 0.01 *
ocular discharge							
B_BAC_ (n = 5)	1.8 ± 0.5	1.8 ± 0.5	*p = 0.5*	1.0 ± 0.0	p < 0.01 *	0.6 ± 0.6	p = 0.02 *
B (n = 9)	1.2 ± 0.8	1.1 ± 0.6	*p = 0.34*	1.0 ± 0.7	*p = 0.08*	0.8 ± 0.4	*p = 0.05*
fluorescein stippling							
B_BAC_ (n = 5)	0.6 ± 0.9	0.8 ± 0.8	*p = 0.19*	0.0 ± 0.0	*p = 0.10*	0.0 ± 0.0	*p = 0.10*
B (n = 9)	1.1 ± 0.8	0.3 ± 0.5	p < 0.01 *	0.0 ± 0.0	p < 0.01 *	0.2 ± 0.4	p < 0.01 *

* statistically significant.

**Table 5:** Mean ± SD of interferometry and meibography grades of the studied dogs at different timepoints (T0, T1, T2, and T3).

	T0		T1			T2			T3		
interferometry											
B_BAC_	3.0 ± 2.1	n = 5	3.3 ± 1.3	n = 5	*p* = 0.5	3.4 ± 1.5	n = 5	*p* = 1.0	3.4 ± 1.5	n = 5	*p* = 1.0
B	1.7 ± 1.2	n = 9	1.8 ± 1.3	n = 9	*p* = 0.09	2.2 ± 1.2	n = 9	*p* = 0.11	2.3 ± 1.3	n = 7	*p* = 0.2
meibography											
B_BAC_	1.6 ± 1.0	n = 7	1.3 ± 0.8	n = 7	*p* = 0.18	1.1 ± 0.8	n = 8	*p* = 0.04 *	1.3 ± 1.0	n = 8	*p* = 0.09
B	1.7 ± 1.1	n = 11	1.8 ± 0.9	n = 12	*p* = 0.04 *	1.2 ± 0.9	n = 13	*p* = 0.04 *	1.7 ± 0.8	n = 13	*p* = 0.5

* statistically significant.

**Table 6:** Mean ± SD of tear meniscus height (in mm) of the studied dogs at different timepoints (T0, T1, T2, and T3).

	B_BAC_				B			
	Mean ± SD	n	95% CI	*p*-Value	Mean ± SD	n	95% CI	*p*-Value
T0	0.58 ± 0.4	n = 15	0.24–0.91	-	0.56 ± 0.4	n = 27	0.28–0.83	-
T1	0.55 ± 0.3	n = 15	0.32–0.77	0.38	0.45 ± 0.2	n = 27	0.30–0.59	0.10
T2	0.51 ± 0.2	n = 15	0.33–0.69	0.35	0.39 ± 0.2	n = 27	0.28–0.51	0.08
T3	0.57 ± 0.3	n = 15	0.32–0.82	0.48	0.58 ± 0.4	n = 21	0.30–0.85	0.46

SD = standard deviation and CI = confidence interval.

**Table 7:** Mean ± SD of VEGF values (in pg/mL) of the studied dogs before (T0) and after treatment (T2).

	B_BAC_				B			
	Mean ± SD	n	95% CI	*p*-Value	Mean ± SD	n	95% CI	*p*-Value
T0	58.3 ± 24.6	n = 3	30.5–86.1	-	50.5 ± 5.7	n = 3	45.5–55.5	-
T2	47.0 ± 7.1	n = 5	39.0–55.0	0.32	57.2 ± 12.7	n = 5	46.0 ± 68.3	0.44

**Table 8:** Mean ± SD of pain scores of the studied dogs at different timepoints (T0, T1, T2, and T3).

		T0	T1	T2	T3
eye comfort	B_BAC_ (n = 5)	1.6 ± 0.89	1.4 ± 0.55	0.8 ± 0.45	0.2 ± 0.45
	B (n = 9)	1.89 ± 0.60	1.0 ± 0.71	0.56 ± 0.0	0.89 ± 0.78
unprovoked behavior	B_BAC_ (n = 4)	0.25 ± 0.50	0.0 ± 0.0	0.0 ± 0.0	0 ± 0
	B (n = 7)	0.14 ± 0.38	0.0 ± 0.0	0.0 ± 0.0	0.0 ± 0.0
interactive behavior	B_BAC_ (n = 5)	1.2 ± 0.45	1 ± 0.72	0.2 ± 0.45	0.6 ± 0.25
	B (n = 9)	1.44 ± 0.73	0.78 ± 0.83	0.53 ± 0.0	0.89 ± 0.60

## Data Availability

The original contributions presented in this study are included in the article/Supplementary Material. Further inquiries can be directed to the corresponding author(s).

## Supplementary Materials

The following supporting information can be downloaded at: https://www.mdpi.com/article/10.3390/vetsci13040388/s1, Table S1: Ocular disease details of the studied dogs; Table S2: Systemic disease details of the studied dogs; Table S3: Pain assessment.

## Abbreviations

The following abbreviations are used in this manuscript:

B	Unpreserved bevacizumab eyedrops
B_BAC_	Preserved bevacizumab eyedrops
BAC	Benzalkonium chloride
CI	Confidence interval
CNV	Corneal neovascularization
CYA	Cyclosporine
DEX	Corticosteroids
DED	Dry eye disease
EDE	Evaporative dry eye
FC	Female castrated
IL	Interleukin
INF	Interferon-gamma
IOP	Intraocular pressure
M	Male
MC	Male castrated
MG	Meibomian gland
MGD	Meibomian gland drop-out
MMP	Matrix metalloproteinase
OD	Os dexter
OS	Os sinister
SD	Standard deviation
STT-1	Schirmer-Tear test 1
TMH	Tear meniscus height
TNF	Tumor necrosis factor
VEGF	Vascular endothelial growth factor
