# Supplementary material for: Topical Application of Preserved and Unpreserved Bevacizumab Eye Drops Improves Ocular Surface Parameters in Dogs with Chronic Keratitis: A Pilot Study
Source: Vet Sci. 2026 Apr 17;13(4):388. doi: 10.3390/vetsci13040388 (PMC13120455; doi:10.3390/vetsci13040388)
Supplement: Supplementary file 1 [file vetsci-13-00388-s001.zip › vetsci-4188933-supplementary.pdf]

Supplemental Table S1. Ocular disease details of the studied dogs.

Eye #	Ocular disease details	CYA (duration of treatment)	DEX (duration of treatment)
	B_{BAC}		
1	Suspected immune-mediated keratitis, normotensive secondary glaucoma (responded well to carbonic anhydrase inhibitors and latanoprost), multiple small lid masses without corneal contact; St.p. immune-mediated blepharitis (responded well to systemic DEX), phacoemulsification and implantation of IOL; Persistent corneal vascularization despite topical CYO, regression when treated with topical bevacizumab, relapse 16 month later	3y 5y 8d[a]	N/A
2	Suspected immune-mediated keratitis, shallow orbita; St.p. distichiasis; Persistent corneal vascularization despite topical CYO and DEX	1y 23d[a]	1y 23d[a]
3	Suspected immune-mediated keratitis, shallow orbita; Persistent corneal vascularization despite topical CYO and DEX	1y 23d[a]	1y 23d[a]
4	Suspected immune-mediated keratitis, spastic entropium; persistent corneal vascularization despite topical CYO and DEX	2m 20d[b]	2m 20d[a]
5	Unilateral keratitis, suspected association with corneal trauma mild distichiasis, macroblepharon, recurrent blepharitis, trichiasis of the caruncle; Persistent corneal neovascularization despite topical CYO	11m 5d[a]	N/A
	B		
6	Stromal bleeding, multiple small lid masses without corneal contact; St.p. blepharitis (responded well to systemic DEX), phacoemulsification and posterior capsulorhexis, excision lid mass; Persistent corneal vascularization despite topical CYO and DEX	3y 5y 8d[a]	N/A
7	Suspected immune-mediated keratitis, spastic entropium; Persistent corneal vascularization despite topical CYO and DEX	2m 20d[b]	2m 20d[a]

8	Suspected immune-mediated keratitis and/or qualitative and quantitative tear deficiency (tear deficiency responded to topical DEX/CYA and systemic pilocarpine, corneal vascularization persisted), ocular brachycephalic syndrome; St.p. canthoplasty due to macroblepharon, cherry eye	1y 1m 28d[b]	1y 7d - discontinued 5m 17d before study recruitment due to corneal degeneration[a]
9	Suspected immune-mediated keratitis and/or qualitative and quantitative tear deficiency (tear deficiency responded to topical DEX/CYA and systemic pilocarpine, corneal vascularization persisted), ocular brachycephalic syndrome; St.p. canthoplasty due to macroblepharon, cherry eye	1y 1m 28d[b]	1y7d - discontinued 5m 17d before study recruitment due to corneal degeneration[a]
10	Suspected immune-mediated keratitis and/or qualitative and quantitative tear deficiency (tear deficiency responded to topical DEX/CYA and systemic pilocarpine, corneal vascularization persisted), trichiasis of caruncle	4y 8m 12d[a]	4y 4m 1d[b]
11	Suspected immune-mediated keratitis and/or qualitative and quantitative tear deficiency (tear deficiency responded to topical DEX/CYA and systemic pilocarpine, corneal vascularization persisted), trichiasis of caruncle	4y 8m 12d[a]	4y 4m 1d[b]
12	Suspected immune-mediated keratitis and/or association with corneal trauma/foreign body, ocular brachycephalic syndrome, C. immatura; St.p. Collagenolytic ulcer treated with CXL and tT, removal of corneal superficial stromal foreign body, DBD due to SCCED; Persistent corneal vascularization despite topical CYO/DEX and bevacizumab (14d treatment period)	4y 20d[b]	4y 20d[a]
13	Unilateral keratitis suspected association with corneal deep stromal collagenolytic ulcer (responded well to CXL and tT) and/or dry eyes (responded well to CYO, corneal neovascularization persisted), distichiasis, ocular brachycephalic syndrome, pigmented conjunctiva, recurrent blepharitis, trichiasis of caruncle	6m 4d[b]	N/A
14	suspected immune-mediated keratitis and/or association with corneal ulcer, lid mass (suspected melanoma), macroblepharon; Persistent corneal neovascularization despite topical CYO/DEX	1y 6m 16d[a]	1y 6m 16d[b]

CYA = cyclosporine A ([a]0.2%, [b]2%)

CXL = Cross-linking

DBD = diamond burr debridement

DEX = dexamethasone ([a]1mg/mL, [b]1mg/g)
SCCED = superficial chronic corneal epithelial defect
St.p. = Status post
tT = temporary tarsorrhaphy
y = year(s); m = month(s); d = day(s)

Supplement Table S2. Systemic disease details of the studied dogs.

Patient	systemic disease	systemic treatment
A *	Food allergy, mitral valve insufficiency, no deficits (accidental finding during routine examination)	Benazepril
B *	none	N/A
C *	Atopic dermatitis	N/A
D	Atopic dermatitis	Intermittent oclacitinib
E *	Atopic dermatitis, orthopaedic problems	Pilocarpin 2%,<br>subdermal hyposensibilization
F *	none	Pilocarpine 1%
G	Atopic dermatitis, histiocytoma left metatarsal region;<br>St.p. palatoplasty and naresplasty	Lokivetmab (every 8 weeks)
H	Mild CKD, IBD, prolapsed intervertebral disc	N/A
I	none	N/A

St.p. = Status post
CKD = Chronic kidney disease
IBD = Inflammatory bowel disease

Supplement Table S3. Modified pain scores system [28].

Pain score category	Description	Pain score
**Unprovoked behavior**	Dog is calm and interested in surroundings	0
	Dog shows mild agitation or is depressed, not interested in surroundings	1
	Dog shows moderate agitation and is restless	2
	Dog is extremely agitated	3
**Eye comfort** indicated by the degree of blepharospasm, blinking, scratching and/or tearing	Eyelids are completely open and in physiological position	0
	Eyelids are partially closed (25 %)	1
	Eyelids are partially closed (50 %); dog shows mild tearing	2
	Eyelids are partially closed (75 %); dog shows moderate tearing	3
	Eyelids are completely closed; dog shows marked tearing	4
**Interactive behavior** indicated by the reaction when the eye surroundings were touched	Normal	0
	Pulls head away when eyes getting touched	1
	Vocalizes when eyes getting touched	2
	Violent reaction to touching of eye (biting, snapping, groaning)	3
